# Systemic Biomarker Alterations in Alcohol and Psychoactive Substance Users: A Cross-Sectional Study

**DOI:** 10.3390/biom16081082

**Published:** 2026-07-24

**Authors:** Lucas A. de Lima Paula, Joice Margareth de A. Rodolpho, Krissia F. Godoy, Juliana A. Prado, Rodrigo Jaccottet Freitas, Carlos Speglich, Fernanda F. Anibal

**Affiliations:** 1 Laboratory of Inflammation and Infectious Diseases, Federal University of São Carlos (UFSCar), São Carlos 13565-905, SP, Brazilffanibal@ufscar.br (F.F.A.); 2 Department of Medicine, Federal University of São Carlos (UFSCar), São Carlos 13565-905, SP, Brazil; 3 Leopoldo Américo Miguez de Mello Research Center CENPES/Petrobras, Rio de Janeiro 21941-915, RJ, Brazil

**Keywords:** alcohol, biomarkers, interleukin-6, substance use disorder, systemic inflammation

## Abstract

Substance use disorders (SUDs) are increasingly associated with systemic inflammatory, neuroendocrine, and cardiometabolic dysregulation; however, the biological signatures underlying distinct substance-use patterns remain incompletely characterized. This study compared biomarker profiles among healthy controls and clinical groups comprising alcohol-associated polysubstance users (A + PS), non-alcohol substance users (PS-A), and alcohol-associated polysubstance users with comorbid post-traumatic stress disorder (PTSD+). Plasma levels of interleukin-6 (IL-6), C-reactive protein (CRP), cortisol, and N-terminal pro-B-type natriuretic peptide (NT-proBNP) were evaluated through comparative, multivariate, dose–response, and receiver operating characteristic (ROC) curve analyses. All clinical groups exhibited significantly increased IL-6, CRP, cortisol, and NT-proBNP levels compared with controls, indicating systemic physiological dysregulation associated with substance exposure. Among the evaluated biomarkers, IL-6 demonstrated the most robust and consistent performance across all analytical approaches. Multivariate regression identified alcohol consumption as an independent predictor of IL-6 elevation (*p* = 0.0003), while dose–response analysis revealed progressive increases in IL-6 according to alcohol consumption severity. ROC analysis further demonstrated that IL-6 exhibited the highest discriminatory performance (AUC = 0.844), outperforming CRP, cortisol, and NT-proBNP in distinguishing substance-using individuals from controls. Collectively, these findings were able to identify IL-6 as a central biomarker associated with substance-related systemic inflammatory dysregulation and support its translational potential as a sensitive indicator of physiological burden associated with chronic alcohol and substance exposure.

## 1. Introduction

Substance Use Disorders (SUDs) represent a major public health challenge and are frequently associated with substantial psychiatric, metabolic, and cardiovascular burden. Recent epidemiological data indicate a marked increase in the prevalence of substance-related disorders worldwide, particularly involving alcohol, marijuana, and opioids [1,2]. In parallel, individuals with psychiatric conditions exhibit disproportionately higher rates of alcohol, tobacco, and illicit drug consumption, reinforcing the complex interaction between substance use and mental health disturbances [3].

According to epidemiology data, the prevalence of substance use disorders among individuals aged ≥12 years increased from 20.3 to 48.4 million cases, with Alcohol Use Disorder (AUD) rising from 14.8 to 27.9 million cases, marijuana use disorder from 4.4 to 20.6 million, and opioid use disorder from 2.0 to 4.8 million cases [4].

Among psychiatric comorbidities, Post-Traumatic Stress Disorder (PTSD) shows a particularly strong association with substance use. Evidence suggests that many individuals consume alcohol or other psychoactive substances as maladaptive coping strategies to attenuate intrusive thoughts, hyperarousal, and emotional distress associated with PTSD [5,6]. Conversely, chronic substance consumption may increase vulnerability to traumatic exposure and worsen psychiatric symptom severity, contributing to a bidirectional cycle of neuropsychological and physiological dysregulation [5,7].

Accumulating evidence indicates that chronic substance exposure promotes persistent activation of inflammatory and stress-related pathways. In addition, it is established that cytokine profiles may vary according to different stages of chronic substance exposure, including active use, withdrawal, and periods of abstinence [8]. Long-term alcohol and drug use have been associated with alterations in adaptive immune responses, increased circulating cytokines, and dysregulation of the hypothalamic–pituitary–adrenal (HPA) axis. Inflammatory mediators such as interleukin-6 (IL-6) and C-reactive protein (CRP), together with neuroendocrine markers including cortisol and cardiometabolic indicators such as N-terminal pro-B-type natriuretic peptide (NT-proBNP), have emerged as promising biomarkers linking systemic inflammation, psychiatric vulnerability, and cardiovascular dysfunction [9,10].

Importantly, chronic low-grade inflammation has been increasingly recognized as a central mechanism underlying the interaction between metabolic dysfunction and neuropsychiatric disorders. Persistent activation of pro-inflammatory signaling pathways may contribute to alterations in neural plasticity, monoaminergic neurotransmission, oxidative stress, and stress-response regulation, thereby influencing behavioral and emotional outcomes associated with substance use [11,12].

Therefore, the present study aimed to compare inflammatory, neuroendocrine, and cardiovascular biomarker profiles among healthy controls and distinct substance-use clinical groups, including alcohol-associated polysubstance use, non-alcohol substance use, and substance use with comorbid PTSD. Additionally, we investigated the ability of IL-6, CRP, cortisol, and NT-proBNP to characterize substance-use patterns and predict substance-associated physiological dysregulation through comparative, multivariate, dose–response, and ROC curve analyses.

## 2. Materials and Methods

### 2.1. Participants and Eligibility

This investigation received approval from the Research Ethics Committee of the Federal University of São Carlos (UFSCar) (protocol no. 54381621.0.0000.5504) and was registered on Plataforma Brasil, the national unified system for research involving human participants within the CEP/Conep framework. All participants provided written informed consent prior to enrollment. Recruitment was conducted through convenience sampling among volunteers receiving care at psychiatric hospitals and Psychosocial Care Centers (CAPS) located in the interior region of São Paulo State, Brazil.

Inclusion criteria comprised individuals aged 18 years or older, of either sex, with a psychiatric diagnosis established according to the International Classification of Diseases, 10th Revision (ICD-10), as documented in their medical records, and without documented comorbid conditions. Exclusion criteria included the presence of cardiovascular disease, autoimmune disorders, inflammatory diseases, chronic systemic illnesses, or diabetes mellitus.

A total of 158 individuals with ICD-10 F10–F19 diagnoses were screened for eligibility. Of these, 57 were excluded due to exclusion criteria, incomplete data or failure to respond to study-related questions. The final analytical sample comprised 101 participants. From these 101 participants three distinct groups were selected to further cross-sectional analysis: alcohol-associated polysubstance users (A + PS, *n* = 48), non-alcohol substance users (PS-A, *n* = 21), and alcohol-associated polysubstance users with comorbid Post-Traumatic Stress Disorder (PTSD+, *n* = 32). Control participants (*n* = 40) were enrolled according to predefined health criteria, including body mass within the normal range, regular engagement in physical activity, adequate sleep quality, absence of tobacco, alcohol or psychoactive substance use, and no history of psychiatric disorders or relevant clinical comorbidities.

To characterize the sample, the following data were collected: presence of a psychiatric diagnosis with medical record documentation according to the ICD-10, age (years), sex, weight (kg), height (cm), body mass index (BMI: kg/m^2^), and clinical follow-up with a psychologist and/or psychiatrist. Participants completed a structured informed consent document which collected demographic information and data regarding physical and mental health status.

Peripheral blood samples were obtained at a single time point for laboratory assessment of inflammatory biomarkers such as C-Reactive Protein (CRP), Interleukin 6 (IL-6), metabolic biomarkers (cortisol), and cardiac biomarker N-terminal pro-B-type Natriuretic Peptide (NT-proBNP).

### 2.2. Procedures and Instruments

Data collection and blood sampling were conducted on-site at the participating psychiatric institutions and Psychosocial Care Centers (CAPS), where a temporary mobile laboratory was established. The assessment protocol included the collection of anthropometric data, depression and anxiety scores, alcohol and psychoactive substance use patterns, and smoking status. Symptoms of depression, anxiety, and stress were assessed using the Depression, Anxiety and Stress Scale (DASS-21), validated for the Brazilian population. The instrument consists of 21 items, with 7 items per subscale, scored on a 4-point Likert scale ranging from 0 to 3, reflecting symptom severity over the previous week. Scores were calculated by summing the corresponding items for each subscale and categorized according to established cut offs. Depression was classified as normal (0–9), mild (10–13), moderate (14–20), or severe (>21). Anxiety was classified as normal (0–7), mild (8–9), moderate (10–14), or severe (≥15). Stress was classified as normal (0–14), mild (15–18), moderate (19–25), or severe (≥26). Additionally, venous blood samples were obtained from each participant for the analysis of metabolic biomarkers, as detailed in Section 2.4.

### 2.3. Alcohol and Psychoactive Substance Consumption Patterns

Alcohol and psychoactive substance consumption were categorized based on the form responses as follows: non-drinkers/users or occasional drinkers/users (with a frequency of up to once a week) and regular drinkers/users (more than once a week).

### 2.4. Blood Collection

A single venous blood sample was obtained from each participant between 7:00 and 10:00 a.m., without fasting requirements. Serum was separated by centrifugation at 4.000× *g* for 10 min. The samples were analyzed for the same biomarkers indicated above. Serum was stored at −80 °C until a one run analysis.

Biomarker (NT-proBNP, CRP) quantification was performed by immunofluorescence using specific commercial kits (Celer, Guangzhou Wondfo Biotech Co., Ltd., Guangzhou, China and In Vitro Diagnostica Ltd., Itabira, Brazil), according to the manufacturers’ instructions. Inflammatory cytokines (IL-6) and cortisol were quantified in serum samples by chemiluminescence using the IMMULITE 1000 system (Siemens, Munich, Germany).

### 2.5. Statistical Analysis

Statistical analyses were performed using GraphPad Prism 9.0 (GraphPad Software, San Diego, CA, USA), and Python 3.12 (with the pandas, scikit-learn, and seaborn libraries). Data normality was assessed using the Shapiro–Wilk test. The normality of variables was assessed using the Shapiro–Wilk test. Continuous variables with a normal distribution were expressed as mean ± standard deviation and compared using a one-way ANOVA. For non-parametric data, the Kruskal–Wallis test was used, followed by Dunn’s post hoc test. Categorical variables were presented as percentages and compared using the chi-square (χ^2^) test. Variables included in the multivariable regression models were selected a priori based on their biological relevance and previously established associations with systemic inflammation and substance use disorders reported in the literature, rather than through automated or data-driven selection procedures. Multivariable regression models were adjusted for potential confounding variables identified a priori based on biological relevance and previous literature, including age, smoking status, underlying diseases, sleep quality, and other clinically relevant covariates. As biomarker distributions were non-Gaussian, data are presented as median and interquartile range (IQR), and nonparametric tests were applied. The significance level for all analyses was set at *p* < 0.05. The discriminatory capacity of biomarkers was evaluated using Receiver Operating Characteristic (ROC) curves, with the area under the curve (AUC) calculated. An AUC value > 0.7 was considered indicative of good accuracy. Given that the ROC analysis represented one of the main analytical approaches of this study, a retrospective sample size assessment was performed according to the methodology proposed by Obuchowski [13]. The required sample size was estimated based on the observed AUC and different predefined margins of error (d = 0.05 and d = 0.10) to evaluate the precision of the ROC estimates.

## 3. Results

### 3.1. Sociodemographic and Behavioral Characteristics Among Healthy Individuals and Substance Use Disorder Groups

A cross-sectional analysis of the participants’ characteristics was conducted with a total of 141 volunteers, distributed into the following groups: control (*n* = 40), A + PS (*n* = 48), PS-A (*n* = 21), and PTSD+ (*n* = 32), as shown in Table 1.

Age showed significant differences between groups (*p* < 0.005), with the A + PS and PS-A groups presenting the highest mean age (approximately 39 years), whereas the control group had the lowest mean age (31.68 ± 9.54 years). In contrast, no significant differences were observed in BMI values, which ranged from approximately 24 to 26 kg/m^2^ across groups. Regarding gender distribution, females predominated only in the control group (55%), whereas the clinical groups were predominantly male (75% to 85.71%).

Concerning risk behaviors, smoking prevalence was high across all clinical groups, ranging from 84.38% in the PTSD+ group to 85.71% in the PS-A group and 85.42% in the A + PS group, suggesting a strong association between alcohol and psychoactive substance use and smoking behavior.

### 3.2. Elevated Inflammatory, Neuroendocrine, and Cardiovascular Biomarkers in Substance-Using Patients

Individuals with SUDs exhibited a consistent and significant elevation across inflammatory (IL-6, CRP), neuroendocrine (cortisol), and cardiovascular (NT-proBNP) biomarkers compared to healthy controls (Table 2).

Comparative analysis of biomarker distributions across clinical groups revealed a consistent pattern of systemic inflammatory and physiological dysregulation associated with substance use. Among all evaluated biomarkers, IL-6 and CRP demonstrated the most robust and homogeneous alterations, showing significantly elevated levels in all substance-use groups compared with healthy controls (*p* < 0.0001 and *p* = 0.0003, respectively), being CRP twice increased in all clinical groups in comparison to control, supporting the presence of sustained systemic inflammation and reinforcing the biological coherence of the IL-6 inflammatory axis (Table 2). Also, median IL-6 and CRP concentrations remained consistently increased across A + PS, PS-A, and individuals with comorbid PTSD, indicating that inflammatory activation is a stable biological feature associated with substance exposure regardless of psychiatric status.

Notably, cortisol concentrations progressively increased across clinical groups, with the highest median values observed in the PTSD+ group (*p* = 0.0094), suggesting cumulative activation of neuroendocrine stress pathways in association with chronic substance exposure and psychiatric burden. On the other hand, NT-proBNP levels only differed (*p* = 0.0021) between control and A + PS groups, with less consistent elevation patterns, indicating possible cardiometabolic involvement in a subset of individuals suffering from AUDs rather than a generalized response.

**Figure 1:**
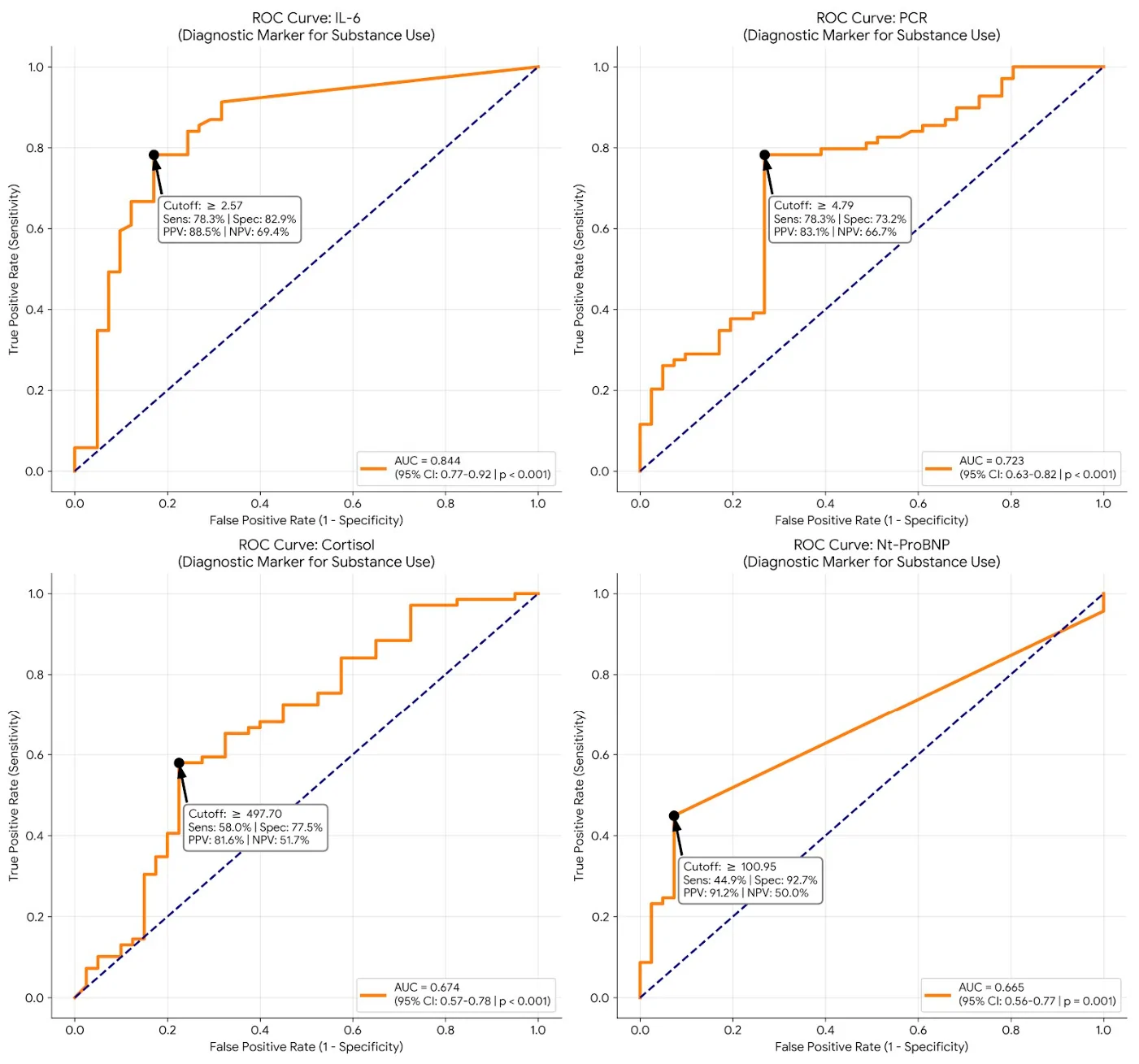
ROC curve analysis of inflammatory, neuroendocrine, and cardiovascular biomarkers associated with substance use. ROC (Receiver Operating Characteristic) curves of IL-6, C-reactive protein (CRP), cortisol, and NT-proBNP were generated to evaluate their discriminatory ability between substance-using individuals and healthy controls. Performance is expressed by the area under the curve (AUC). The orange solid line represents the ROC curve for each biomarker comparison, indicating diagnostic performance (sensitivity vs. 1 − specificity). The dashed diagonal line represents the line of no discrimination (AUC = 0.50), indicating the performance expected by random classification. Optimal cutoff values, sensitivity, and specificity are indicated in each graph.

In order to evaluate each biomarker related with substance consumption, ROC curves were generated (Figure 1); the analysis demonstrated distinct discriminatory performances among the investigated biomarkers.

Among the evaluated markers, IL-6 demonstrated the best balance between sensitivity and specificity, supporting its potential utility as a biomarker of substance-associated systemic inflammation. IL-6 exhibited the highest predictive accuracy, with an AUC of 0.844, indicating strong discriminative ability and the best balance between sensitivity (0.78) and specificity (0.83) at the optimal cutoff value (≥2.57). This higher discriminatory capacity points toward a real systemic inflammation as it increases consistently among the substance users, emerging as a potential translational biomarker as it could differentiate exposed individuals. Meanwhile, CRP demonstrated moderate discriminatory performance (AUC = 0.723), with balanced sensitivity and specificity values (0.78 and 0.73, respectively), supporting its utility as a secondary marker of systemic inflammation associated with SUDs. In contrast, cortisol (AUC = 0.674) and NT-proBNP (AUC = 0.665) showed lower predictive performance, implying more limited discriminatory capacity. Although NT-proBNP presented high specificity (0.93), its low sensitivity (0.45) indicates reduced effectiveness as a screening biomarker.

### 3.3. Dose–Response and Predictive Value of IL-6 in Relation to Substance Use Pattern

To investigate the association across substance use patterns and systemic inflammation, a multivariate approach was applied using ordinal exposure scales and log_10_-transformed biomarker data to address skewed distributions. Substance consumption variables (alcohol, tobacco, and drugs) were categorized into ordinal levels reflecting increasing exposure, enabling dose–response assessment. Multiple linear regression models were performed for each biomarker to estimate the independent effects of each substance on inflammatory markers (Table 3).

**Figure 2:**
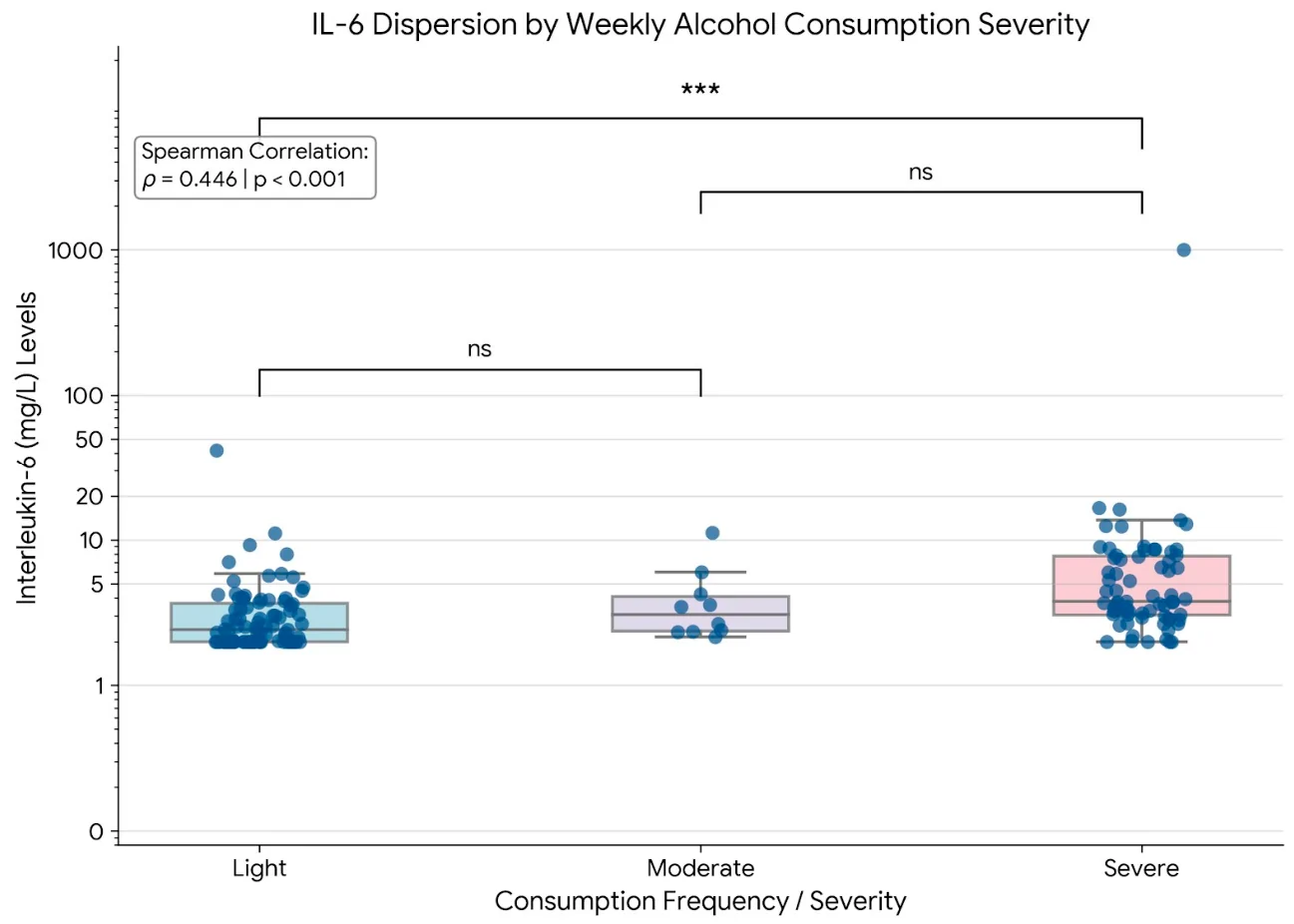
Dose–response relationship between alcohol consumption and IL-6 levels. Log_10_-transformed IL-6 concentrations are shown across increasing levels of alcohol consumption (sporadic, moderate weekly, and elevated weekly). Data are presented as individual values with median and interquartile range. A progressive increase in IL-6 levels is observed with higher alcohol intake, consistent with a dose–response pattern. Asterisks indicate statistically significant differences (*** *p* < 0.001), whereas ns indicates a non-significant difference (*p* ≥ 0.05). Colors are used solely to distinguish the study groups and do not indicate statistical significance.

Alcohol consumption exhibited a significant and independent association with IL-6 levels (*p* = 0.0003). The regression coefficient in the log-transformed scale (β = 0.1045) indicates that each incremental increase in alcohol consumption category was associated with an approximate 27.2% elevation in baseline IL-6 levels, supporting a clear dose–response relationship, with an indication that even moderate increases in alcohol intake may contribute to systemic inflammatory activation (Table 3, Figure 2). In regard to tobacco, smoking showed a positive but non-significant association with IL-6 (*p* = 0.0736). The estimated effect size suggests an approximate 17.6% increase in IL-6 levels per escalation in smoking intensity. Although this association did not reach conventional statistical significance, the observed trend indicates a potential dose-dependent effect that may become significant in larger cohorts, reinforcing the biological plausibility of smoking as a contributor to inflammatory burden. In contrast, no associations were observed for other drug use or PTSD status, indicating that alcohol is the primary driver of IL-6 variability.

As observed at Figure 2, a clear dose–response relationship was noted between alcohol consumption and IL-6 levels. As alcohol ingestion increased from sporadic to moderate and elevated weekly use, IL-6 concentrations showed a progressive rise, indicating a consistent escalation in systemic inflammation. Despite interindividual variability, the overall trend remained robust across groups, supporting a graded association between alcohol exposure and inflammatory activation, reinforcing alcohol consumption as an independent contributor to increased IL-6 levels.

**Figure 3:**
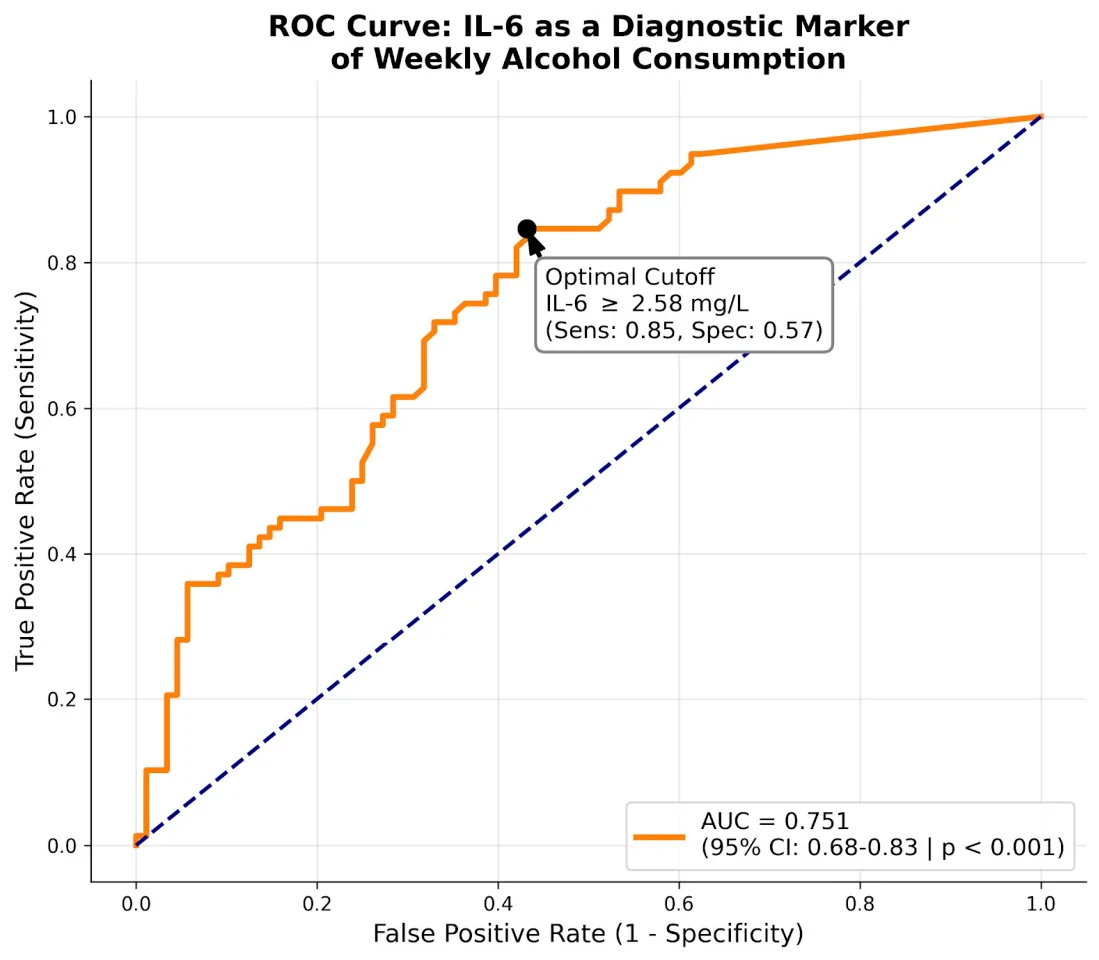
ROC curve analysis of IL-6 as a predictor of weekly alcohol consumption. The ROC (Receiver Operating Characteristic) curve of IL-6 was generated to evaluate its discriminatory ability in identifying individuals with weekly alcohol consumption. Performance is expressed by the area under the curve (AUC = 0.751). The orange solid line represents the ROC curve, indicating the diagnostic performance of IL-6 (sensitivity vs. 1 − specificity), while the blue dashed line represents the reference line (line of no discrimination, AUC = 0.5), corresponding to a test with no discriminatory ability. The optimal cutoff value identified was IL-6 ≥ 2.58, yielding a sensitivity of 85% and a specificity of 57%.

Consistent with the observed dose–response relationship, ROC curve analysis (Figure 3) further demonstrated the discriminative capacity of IL-6 in identifying individuals with higher alcohol consumption. IL-6 exhibited moderate predictive performance (AUC = 0.751), with an optimal cutoff value (≥2.58) yielding high sensitivity (0.85) and moderate specificity (0.57). These findings demonstrate that, beyond reflecting a graded increase in systemic inflammation, IL-6 also possesses practical utility as a biomarker for distinguishing patterns of alcohol use, particularly as a sensitive indicator of elevated consumption.

## 4. Discussion

SUDs represent a major public health concern due to their wide impact on physical health, psychological status, and social functioning, being strongly associated with increased risk of both acute and chronic medical conditions. Chronic alcohol exposure has been linked to immune dysregulation and sustained inflammatory activation, suggesting that chronic inflammation may contribute to the pathophysiology of AUD. In this context, elevated levels of multiple inflammatory biomarkers have been reported in individuals with AUD [14,15].

The present study demonstrated an arrangement of inflammatory, neuroendocrine, and cardiovascular biomarker alterations among individuals with SUDs, reinforcing the concept that these disorders are associated with broad physiological dysregulation extending beyond behavioral and psychiatric manifestations. Comparative analyses across the clinical groups revealed significantly elevated IL-6 (*p* < 0.0001) and CRP (*p* < 0.001) levels in substance-using and PTSD individuals relative to healthy controls, accompanied by progressive increases in cortisol (*p* < 0.01) concentrations and differences in NT-proBNP (*p* < 0.01) levels. These results are aligned with past evidence indicating that SUDs are associated with chronic inflammatory activation, neuroimmune dysfunction, and systemic physiological burden [1,8,16]. Prior studies have consistently reported elevated peripheral cytokines in alcohol-related disorders, particularly IL-6, TNF-α, IL-8, IL-10, IL-12, and CRP, supporting the notion that inflammatory dysregulation is a recurrent biological feature in substance exposure and dependence states [8,17,18,19,20].

Among all evaluated biomarkers, IL-6 emerged in this current investigation as the most robust and homogeneous biological alteration. Elevated IL-6 concentrations were observed across all clinical groups, including isolated psychoactive substance users, combined alcohol and psychoactive substance users, and individuals with positive status to PTSD. This consistent elevation regardless of psychiatric status suggests that inflammatory activation represents a stable physiological characteristic associated with substance exposure itself. Igue and coworkers [21] evaluated 29 patients with psychiatric and substance use disorders, including cannabis, alcohol, and cocaine consumption, and observed significantly elevated circulating levels of interleukins, including IL-6, compared with healthy controls. Supporting this data, Keen et al. [22] and Levandowski et al. [23] analyzed several cocaine and marijuana users founding significantly higher blood IL-6 levels. Together, these observations converge with our findings to demonstrate a close relationship between elevated IL-6 blood levels and individuals suffering from SUDs. Earlier investigations also associated elevated IL-6 levels with cognitive impairment, psychiatric symptoms, withdrawal severity, craving, and alcohol dependence severity, reinforcing the broad biological relevance of this cytokine within substance-related disorders [5,18,20,23,24].

A major input of this study was the identification of IL-6 as the biomarker with the greatest discriminative capacity for substance exposure. ROC curve analyses demonstrated that IL-6 achieved the highest predictive performance among all evaluated biomarkers, with an AUC of 0.844 and a balanced combination of sensitivity and specificity. In contrast, CRP demonstrated moderate discriminatory performance, whereas cortisol and NT-proBNP showed lower predictive capacity. NT-proBNP levels also differed significantly between groups, although with greater variability and lower discriminative performance compared with IL-6. Prior studies have described NT-proBNP as a sensitive marker of cardiac dysfunction, cardiovascular risk, and chronic heart failure severity, while prolonged alcohol exposure has been associated with myocardial damage and elevated natriuretic peptide levels [25,26]. In the present study, the greater variability and lower sensitivity of NT-proBNP suggest that cardiovascular involvement may occur in a more heterogeneous or subgroup-specific manner rather than representing a generalized physiological response across all substance users. Once more, these data position IL-6 as the most consistent biomarker within the evaluated physiological domains and support its potential utility as a translational biomarker capable of distinguishing exposed individuals from healthy controls. Although elevated IL-6 levels have previously been reported in individuals with SUDs [27,28,29], most available studies have focused on isolated patient populations or single inflammatory markers. In contrast, the present study simultaneously evaluated inflammatory, neuroendocrine, and cardiovascular biomarkers across distinct substance-use profiles using complementary analytical approaches, including multivariable regression, dose–response analysis, and ROC curve analysis. This integrated approach allowed us to compare the relative performance of different biomarker classes within the same cohort and demonstrated that IL-6 consistently showed the strongest association with substance use. Rather than establishing the clinical utility of IL-6, these findings support its role as a promising candidate biomarker and provide a rationale for future longitudinal and multicenter studies aimed at validating its diagnostic and prognostic value.

The simultaneous increase in CRP further strengthens the inflammatory profile identified in the present study. Since CRP is described as an acute-phase protein induced primarily by IL-6 [30,31], the parallel elevation of both biomarkers supports the biological coherence of the inflammatory axis observed across the clinical groups. CRP has been associated with alcohol use disorder, excessive alcohol consumption, withdrawal states, stress exposure, and chronic low-grade inflammation [16,32,33]. The association between alcohol intake and CRP levels remains an active area of investigation. In a large cross-sectional study including 2833 participants, moderate alcohol consumption was associated with the lowest circulating CRP concentrations, whereas both lower and very high consumption patterns showed comparatively increased levels, suggesting a potential non-linear relationship between alcohol exposure and systemic inflammatory status [34,35]. Combined, the elevated IL-6 and CRP levels fortify the presence of sustained systemic inflammation associated with substance use patterns.

Another central contribution of the ongoing research was the demonstration of a clear dose–response relationship between alcohol consumption and IL-6 levels. Multivariate regression analyses revealed that alcohol consumption was independently associated with increased IL-6 concentrations, with each incremental increase in alcohol exposure category corresponding to an approximate 27.2% elevation in IL-6 levels. In parallel, graphical dose–response analyses demonstrated a progressive increase in IL-6 concentrations from sporadic alcohol consumption to moderate and heavy weekly drinking patterns. Reports given by Adams et al., [8]; Karoly et al., [28], and Albert et al., [34] are consistent with what we found demonstrating positive associations between alcohol use and inflammatory biomarkers, particularly IL-6 and CRP. Anterior investigations also suggested that the intensity of alcohol exposure may influence inflammatory activation, including studies describing associations between alcohol risk categories and circulating inflammatory markers [29,36,37].

Importantly, although tobacco use demonstrated a positive association trend with IL-6 levels, this relationship did not achieve statistical significance in the present study. Likewise, no independent associations were observed for other drug use or PTSD status in the multivariate analyses, indicating that alcohol consumption represented the principal factor associated with IL-6 variability within the evaluated exposure model. This observation is particularly relevant considering that combined alcohol and cannabis use suggested that cannabis may moderate the relationship between alcohol consumption and IL-6 levels [31]. In the present analysis, however, alcohol remained the primary exposure variable associated with systemic inflammatory activation.

Beyond inflammatory alterations, the data here presented also support the involvement of neuroendocrine and cardiovascular pathways in substance-related physiological dysregulation. Cortisol concentrations progressively increased across the clinical groups, with the highest median values observed among PTSD-positive individuals which are consistent with past evidence indicating that alcohol and other substances influence biomarkers of the hypothalamic–pituitary–adrenal axis and stress-related physiological responses [38,39]. Moreover, Stephens & Wand [40], and Chang and coworkers [41] have reported both blunted cortisol stress responses and elevated basal cortisol levels in heavy alcohol drinkers and individuals with substance dependence, suggesting substantial neuroendocrine heterogeneity within these populations.

Lastly, a limitation of this study was the relatively modest sample size, which primarily resulted from the strict eligibility criteria adopted to recruit participants into well-defined clinical subgroups (A + PS, *n* = 48; PS-A, *n* = 21; and PTSD+, *n* = 32). Given the heterogeneous patterns of substance use and the frequent presence of psychiatric and medical comorbidities among individuals with substance use disorders, identifying participants who met the predefined classification criteria was challenging. Although this approach reduced clinical heterogeneity and strengthened the validity of comparisons across groups, it also resulted in relatively small subgroup sizes, particularly in the PS-A group. Post hoc analyses indicated that the unequal group sizes had only a minimal impact on statistical power for the principal analyses, especially for IL-6; however, some comparisons involving smaller subgroups showed reduced statistical power for certain biomarkers and should therefore be interpreted with caution.

## 5. Conclusions

Our findings indicate that SUDs are associated with a pattern of systemic inflammatory, neuroendocrine, and cardiometabolic alterations, with IL-6 consistently showing the strongest association across the analytical approaches employed. IL-6 levels were significantly higher in all clinical groups, remained independently associated with alcohol consumption after adjustment for potential confounders, increased according to the frequency of substance use, and demonstrated the highest discriminatory performance among the biomarkers evaluated. Elevated CRP levels further support the presence of systemic inflammation, whereas alterations in cortisol and NT-proBNP suggest concomitant neuroendocrine and cardiovascular involvement. Although these findings highlight IL-6 as a promising candidate biomarker for distinguishing individuals with SUDs, its clinical applicability should be confirmed in larger, multicenter, and prospective studies before routine implementation.

## Figures and Tables

**Table 1:** Descriptive analysis of personal data and lifestyle of participants.

Variables		Control (*n* = 40)	A + PS (*n* = 48)	PS-A (*n* = 21)	PTSD+ (*n* = 32)	*p*-Value
Age (M/SD)		31.68 ± 9.54	39.31 ± 9.82	39.33 ± 14.20	38.03 ± 9.77	0.0040 *
BMI (kg/m^2^) (M/SD):		25.77 ± 4.04	24.07 ± 4.73	26.28 ± 3.81	24.99 ± 5.14	0.2162
Gender	Masculine: Feminine:	18 (45%) 22 (55%)	36 (75%) 12 (25%)	18 (85.71%) 3 (14.29%)	24 (75%) 8 (25%)	0.0022 *
Smoking	Non-smoker: Smoker:	40 (100%) 0 (0%)	7 (14.58%) 41 (85.42%)	3 (14.29%) 18 (85.71%)	5 (15.62%) 27 (84.38%)	<0.0001 ****
Alcoholism	Non-drinker: Regular drinker:	40 (100%) 0 (0%)	0 (0%) 48 (100%)	21 (100%) 0 (0%)	0 (0%) 32 (100%)	<0.0001 ****
Psychoactive users	Non-user: Regular user:	40 (100%) 0 (0%)	0 (0%) 48 (100%)	0 (0%) 21 (100%)	0 (0%) 32 (100%)	<0.0001 ****

BMI: Body Mass Index. Data are presented as mean ± standard deviation (M ± SD) for continuous variables and percentages for categorical variables. One-way ANOVA and chi-square tests were used to assess statistically significant differences for continuous and categorical data, respectively. *p*-values listed in the table indicate overall statistical differences between groups (* *p* < 0.05; **** *p* < 0.0001).

**Table 2:** Comparative Analysis of Inflammatory, Neuroendocrine, and Cardiovascular Biomarkers across Substance Use Clinical Groups.

Biomarker	Control (N = 40)	A + PS (N = 48)	PS-A (N = 21)	PTSD+ (N = 32)	*p*-Value
IL-6 (pg/mL)	2.00 [2.00–2.31]	3.44 [2.57–4.74]	3.49 [2.66–6.60]	3.18 [2.62–4.83]	<0.0001
CRP (mg/L)	2.54 [0.83–5.01]	5.00 [5.00–11.26]	5.00 [4.53–7.70]	5.00 [4.45–7.70]	0.0003
Cortisol (nmol/L)	394.52 [232.04–485.96]	506.50 [352.41–637.12]	530.78 [406.62–642.60]	558.07 [378.52–717.02]	0.0094
Nt-ProBNP (pg/mL)	100.00 [100.00–100.00]	112.90 [100.00–164.90]	100.00 [100.00–217.10]	100.00 [100.00–261.98]	0.0021

Alcohol + psychoactive substance users (A + PS), non-alcoholic psychoactive substance users (PS-A) and alcohol + psychoactive substance users with a positive Post-Traumatic Stress Disorder (PTSD+). Data are presented as median (IQR). Comparisons among groups were performed using the Kruskal–Wallis test followed by Dunn’s multiple comparisons test.

**Table 3:** Multiple Linear Regression Model for Predictors of IL-6.

Predictor/Adjustment Factor	Adjusted Coefficient (β)	Estimated Change in IL-6 (%)	95% CI of Change (%)	*p*-Value
**Main Predictors (Substances)**				
Alcohol Consumption (Ordinal Scale)	0.1045	+27.2%	+11.8% to +44.6%	0.0003
Tobacco Use (Ordinal Scale)	0.0704	+17.6%	−1.6% to +40.4%	0.0736
Other Drug Use (Ordinal Scale)	−0.0061	−1.4%	−7.9% to +5.6%	0.6893
**Clinical and Psychiatric Adjustment Factors**				
Positive PTSD Status	−0.0505	−11.0%	−32.8% to +17.9%	0.4148

β coefficients were estimated using multivariable linear regression. The model was adjusted for age, smoking status, underlying diseases, sleep quality, and other clinically relevant covariates selected a priori based on biological relevance. CI, confidence interval.

## Data Availability

The original contributions presented in this study are included in the article. Further inquiries can be directed to the corresponding authors.
